# Comparison of three kinds of self-expandable metallic stents induced granulation tissue hyperplasia in the rabbit trachea

**DOI:** 10.1038/s41598-021-02573-9

**Published:** 2021-11-30

**Authors:** Yahua Li, Mengde Li, Xiaofeng Wang, Yuhui Wang, Chang Li, Yanan Zhao, Zhaonan Li, Jianjian Chen, Jing Li, Kewei Ren, Zongming Li, Jianzhuang Ren, Xinwei Han, Qian Li

**Affiliations:** 1 Department of Interventional Radiology, The First Affiliated Hospital of Zhengzhou University, Zhengzhou, Henan China; 2 Interventional Treatment and Clinical Research Center of Henan Province, Zhengzhou, Henan China; 3 School of Mechanics and Engineering Science, National Center for International Research of Micro-Nano Molding Technology, Zhengzhou University, Zhengzhou, Henan China; 4 Department of Clinical Laboratory, The Third Affiliated Hospital of Zhengzhou University, Zhengzhou, Henan China; 5 Department of Nose, The First Affiliated Hospital of Zhengzhou University, Zhengzhou, Henan China; 6 Interventional Institute of Zhengzhou University, Zhengzhou, Henan China

**Keywords:** Preclinical research, Experimental models of disease

## Abstract

To compare stent-induced granulation tissue hyperplasia of bare (SEMS), polyurethane-covered (PU-SEMS) and electrospun nanofibre-covered (EN-SEMS) self-expandable metallic stents in the rabbit trachea. Twenty-seven rabbits were randomly assigned to 3 groups that received SEMS, PU-SEMS or EN-SEMS. Computed tomography and sacrifice were performed as scheduled. Haematoxylin–eosin and Masson’s trichrome staining protocols were performed for pathological analysis. The data for tracheal ventilation area ratio, qualitative histological scoring, number of epithelial layers, and thicknesses of papillary projection and submucosa were documented and statistically analysed. All stents were successfully placed under the guidance of fluoroscopy without complications. Post-stenting 3 and 7 days, computed tomography revealed that the fully expandable EN-SEMS was similar to the SEMS and PU-SEMS. The mean stented tissue score in the SEMS group was higher than those of both the PU-SEMS and EN-SEMS groups at 3 days post-stenting. The pathological findings suggested that there was no papillary projection formation 3 days after stent placement. The thickness of papillary projection in the SEMS group was significantly higher than those of the PU-SEMS and EN-SEMS groups at 7 days post-stenting. After stenting 4 weeks, the tracheal ventilation area ratio of SEMS, PU-SEMS and EN-SEMS was 0.214 ± 0.021, 0.453 ± 0.028 and 0.619 ± 0.033, respectively. There were significant between-group differences. In conclusion, the stent-induced granulation tissue formation in EN-SEMS is less severe than that of PU-SEMS and SEMS. EN-SEMS has smaller radial force, and the tracheal ventilation ratio after stent placement better than that of PU-SEMS.

## Introduction

Self-expandable metallic stents (SEMS) have been utilized in airways for several decades^1^. SEMS-related granulation hyperplasia is one of the main complications after stent placement, which limits its application in benign indications. Up to now, the U.S. Food and Drug Administration has warned the application of bare SEMS in benign stenoses due to the potential complications related to non-removability^2^. Only temporary use was advised in the application of covered SEMS.

To reduce the granulation hyperplasia and non-removability of SEMS in the airway, many functional tracheal stents and drug-eluting stents, including I^125^ seed-loaded SEMS, biodegradable metallic stents and biodegradable polymer stents, have been developed^3–8^. However, none of them provide perfect care based on the needs of the ideal airway stent^9^. In the study of Wang^3^, a paclitaxel drug‑eluting stent (DES) with a backbone of poly (lactic‑co‑glycolic acid) (PLGA) was designed. The drug release could last up to 5 months. Although the application of DES in a canine model demonstrated that little granulation tissue growth occurred, papillary projection also grew through the mesh of the stent. The application of I^125^ seed-loaded SEMS in healthy beagle dog trachea is feasible and safe, and tracheal stenosis became gradually severe, not associated with I^125^ seeds^6^. Furthermore, local erosions and radiation injury were observed. Luffy^5^ and colleague explored Mg, AZ31, and Mg-3Y alloys for degradable tracheal stent assessment. All alloys resulted in granulation tissue growth around the stent, leading to encapsulation with increasing time. The comparison of balloon-expandable poly-l-lactic acid (PLLA) biodegradable stent (BEBS) and bare SEMS indicated that the degrees of stent-induced granulation tissue hyperplasia were similar, but BEBS was associated with a larger granulation tissue area.

From the development experience of cardiovascular and oesophageal stents, drug-eluting covered metallic stents are promising. To combine the advantages of drug-eluting stents and covered stents, we developed an electrospun nanofibre-covered metallic stent. This kind of nanofibre membrane has the ability to carry drugs, thus offering the potential to achieve local drug delivery. In this study, we compared the granulation hyperplasia caused by bare SEMS, polyurethane-covered SEMS and electrospun nanofibre-covered SEMS in rabbit trachea.

## Material and methods

### Fabrication of EN-SEMS

SEMS and polyurethane covered self-expandable metallic stent (PU-SEMS) were purchased from Nanjing Micro-tech Co., Ltd.. The length and diameter of the stents were 20 mm and 8 mm. SEMSs were fabricated with a single NiTi alloy wire. While PU-SEMSs were coated PU by immersion coating method. The fabrication of the electrospun nanofibre-covered self-expandable metallic stent (EN-SEMS) was achieved using an electrospinning system with 20 kV voltage, under the ambient temperature of 25 degrees Celsius and 30% humidity. The distance between the syringe tip and the collector was 18 cm. Poly(L-lactide-co-caprolactone) (PLCL, lactic acid:caprolactone ratio = 1:1, intrinsic viscosity = 3.9 dL/g) was dissolved in a mixture of dichloromethane and N,N-dimethylformamide with a ratio by volume of 7:3 at a concentration of 5% (w/v) to obtain the electrospinning solution. A total of 1.8 mL solution was supplied for every EN-SEMS.

### Compression test

The radial strength of SEMS, PU-SEME and EN-SEMS were evaluated with parallel plates compression test. Universal testing machine with a 5-pound load cell was used. The displacement control mode with a compression distance of 5 mm was adopted. The compression displacement rate is 0.1 mm/s, which ensures quasi-static. The compression process was monitored to make sure there was no slip between the stent and the plates. The resistance force was recorded during the compression. The radial strength was defined as the resistance force at the compression displacement of 2 mm. The normalized radial strength was calculated as the radial strength divided by the stent length.

### Animal study

**Figure 1:**
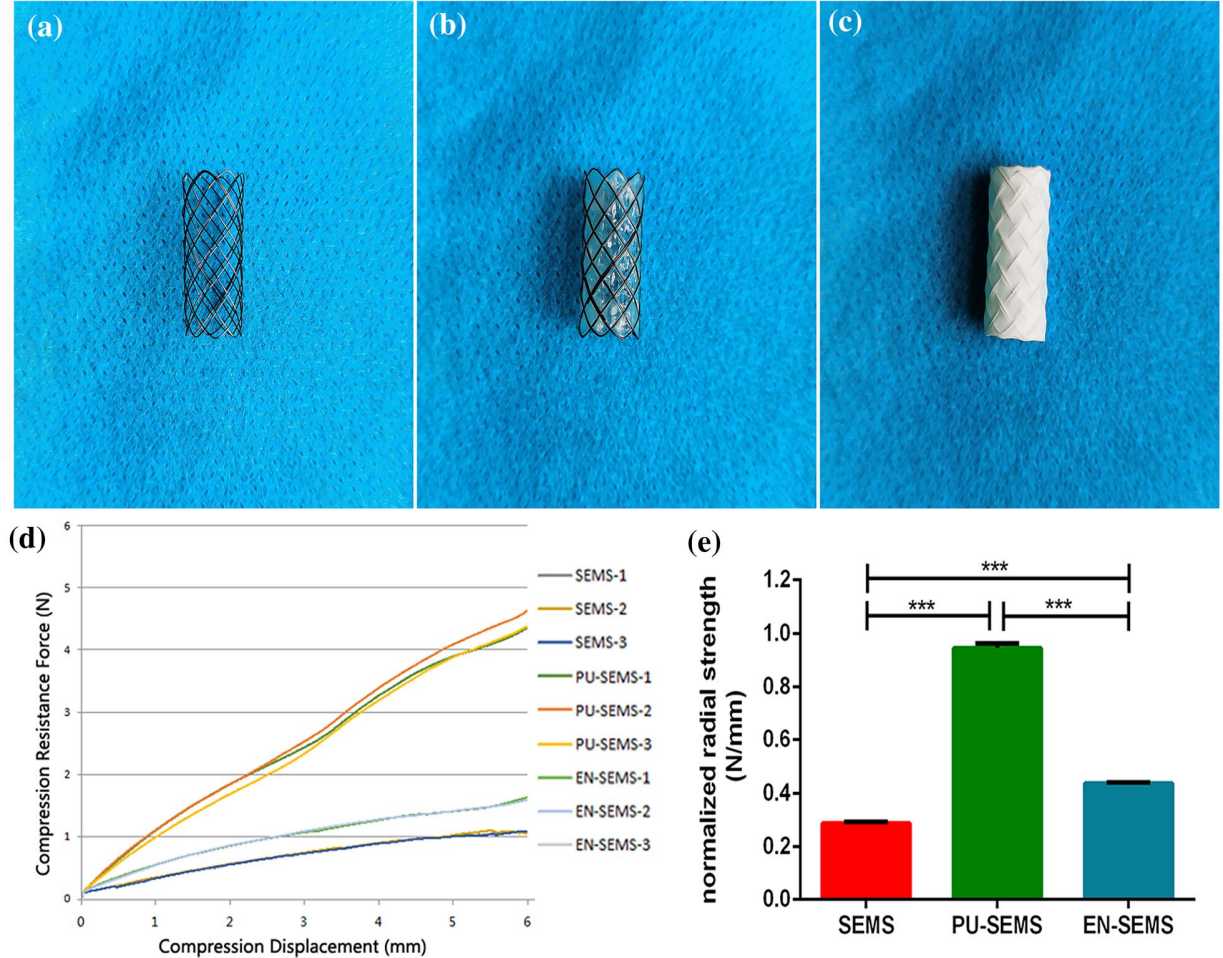
General view of stents and compression test. (**a**–**c**) Representative image of SEMS, PU-SEMS and EN-SEMS. (**d**) Force–Displacement image of compression test. (**e**) Normalized radial strength of SEMS, PU-SEMS and EN-SEMS. P-values < 0.05 (*), < 0.01 (**), < 0.001 (***), and < 0.0001 (****).

This animal study was approved by the Committee on the Ethics of Animal Experiments of Zhengzhou University and reported in accordance with ARRIVE guidelines. All methods were performed in accordance with the relevant guidelines and regulations. Animal studies were conducted at Henan Key Laboratory for Pharmacology of Liver Disease. A total of 27 New Zealand rabbits (both male and female) (2.5 ± 0.2 kg) were assigned in three groups, including a SEMS group (n = 9), a PU-SEMS group (n = 9) and an EN-SEMS group (n = 9) (Fig. 1).

The stents were placed in the trachea under the guidance of fluoroscopy through a delivery system. All rabbits were maintained in single cages at a room temperature of 22 ± 2 °C, a relative humidity of 45 ± 15% and a 12-h light/dark cycle. Standard food and water could be accessed freely. Computed tomography was performed to evaluate stent expansion and granulation hyperplasia at the post-stenting time points of 3 days, 7 days and 4 weeks. The tracheal ventilation area (TVA) was measured by Image J. The TVA ratio was defined as proximal stent part ventilation area divided by middle stent part ventilation area. The rabbits were sacrificed after CT examination was completed. The trachea with stents were sectioned transversely and fixed in 10% neutral buffered formalin for 24 h. Paraffin blocks with trachea were cut in 5-μm-thick sections to obtain slides. Haematoxylin–eosin (HE) and Masson’s trichrome (MTS) staining protocols were performed for pathological analysis.

### Histological analysis

For histological analysis of stented trachea, the number of epithelial layers, the thickness of papillary projections and the thickness of the submucosa were documented. Qualitative histological scoring was performed using a stented tissue scoring system modified from the combination the histopathological scoring system of Ruegemer^10^, Debiane^11^ and Kim^8^ (Table 1). The degree of inflammatory cell infiltration was subjectively judged based on the distribution and density of inflammatory cells. When single leukocyte infiltration was occasionally observed, it was evaluated as mild and scored 0. When the leukocytes had patchy infiltration, it was judged as mild to moderate and scored 1. When leukocytes were aggregated and individuals could not be distinguished, it was regarded as moderate and scored 2. When there was diffuse infiltration of leukocytes throughout the submucosal layer, the degree of moderate to severe was documented, which was scored 3. When there was diffuse infiltration with multiple necrotic foci, the degree of severe was determined, and a score of 4 was given. Collagen deposition was also subjectively determined, where 0 indicated mild; 1, mild to moderate; 2, moderate; 3, moderate to severe; and 4, severe.

**Table 1:** Stented tissue scoring system.

Paremeters	0	1	2	3	4
Cilial loss	Absent	Focal	Moderate	Diffuse	
Surface epithelial changes	Absent	Hyperplasia	Squamous metaplasia	Epithelial loss	
Epithelial erosion	Absent	Epithelial attenuation	1 area of ulceration	2–3 areas of ulceration extensive	≥ 4 areas of ulceration t
Degree of inflammatory cell infiltration	Mild	Mild-moderate	Moderate	Moderate -severe	Severe
Granulation tissue	Absent	Focal	Moderate	Small polyps	Large polyps
Cartilage changes	Absent	Focal	Moderate	Focal necrosis	Extensive necrosis
Collagen deposition	Mild	Mild-moderate	Moderate	Moderate -severe	Severe

### Statistical analysis

Differences between groups were analysed using analysis of variance. Post hoc comparisons were performed using the Bonferroni method. A p value less than 0.05 was considered statistically significant. Statistical analysis was performed using SPSS 21.0 software.

## Results

### Compression test results

The representative compression behavior of SEMS, PU-SEME and EN-SEMS were demonstrated in Fig. 1. The experiment showed a good repeatability for all the three stents. The PU-SEME exhibited a higher radial resistance than SEMS and EN-SEMS. The EN-SEMS exhibited a higher radial resistance than SEMS. The normalized radial strength for all the stents is shown in Figure. The SEMS had less radial strength than PU-SEME and EN-SEMS. The EN-SEMS had less radial strength than PU-SEME. The normalized radial strength in SEMS, PU-SEMS and EN-SEMS were 0.287 ± 0.004, 0.945 ± 0.017 and 0.437 ± 0.004 N/mm respectively.

### Stent placement

**Figure 2:**
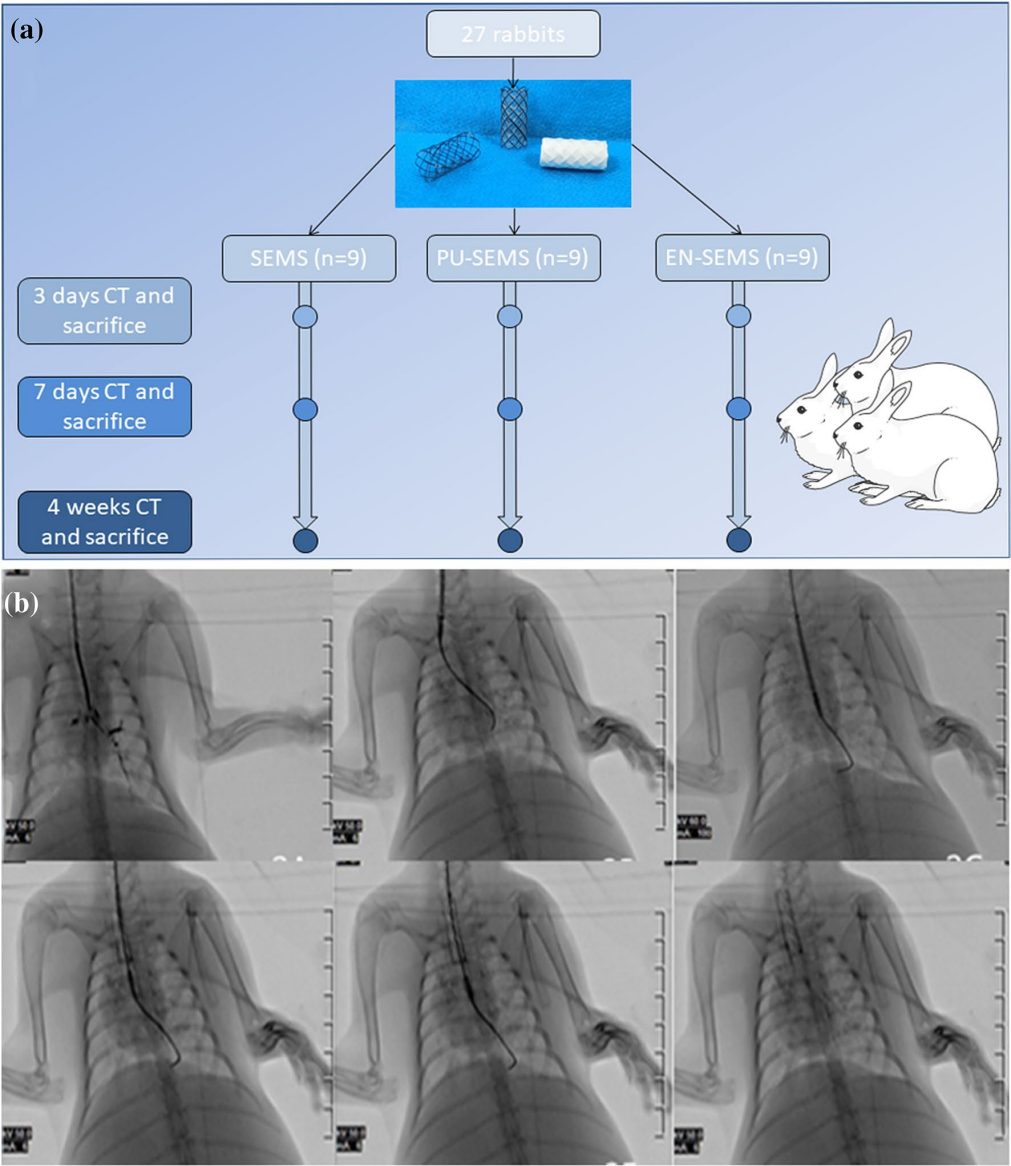
Flow chart of study design and stent placement. (**a**) Flow chart showing the process and follow-up. (**b**) The process of stent placement under the guidance of fluoroscopy.

A total of 27 stents (9 stents in each group) were successfully implanted in 27 rabbits (Fig. 2). The stents were released by a 5F delivery system under the guidance of fluoroscopy. No procedure-related haemoptysis or pneumothorax occurred.

### Computed tomography findings

**Figure 3:**
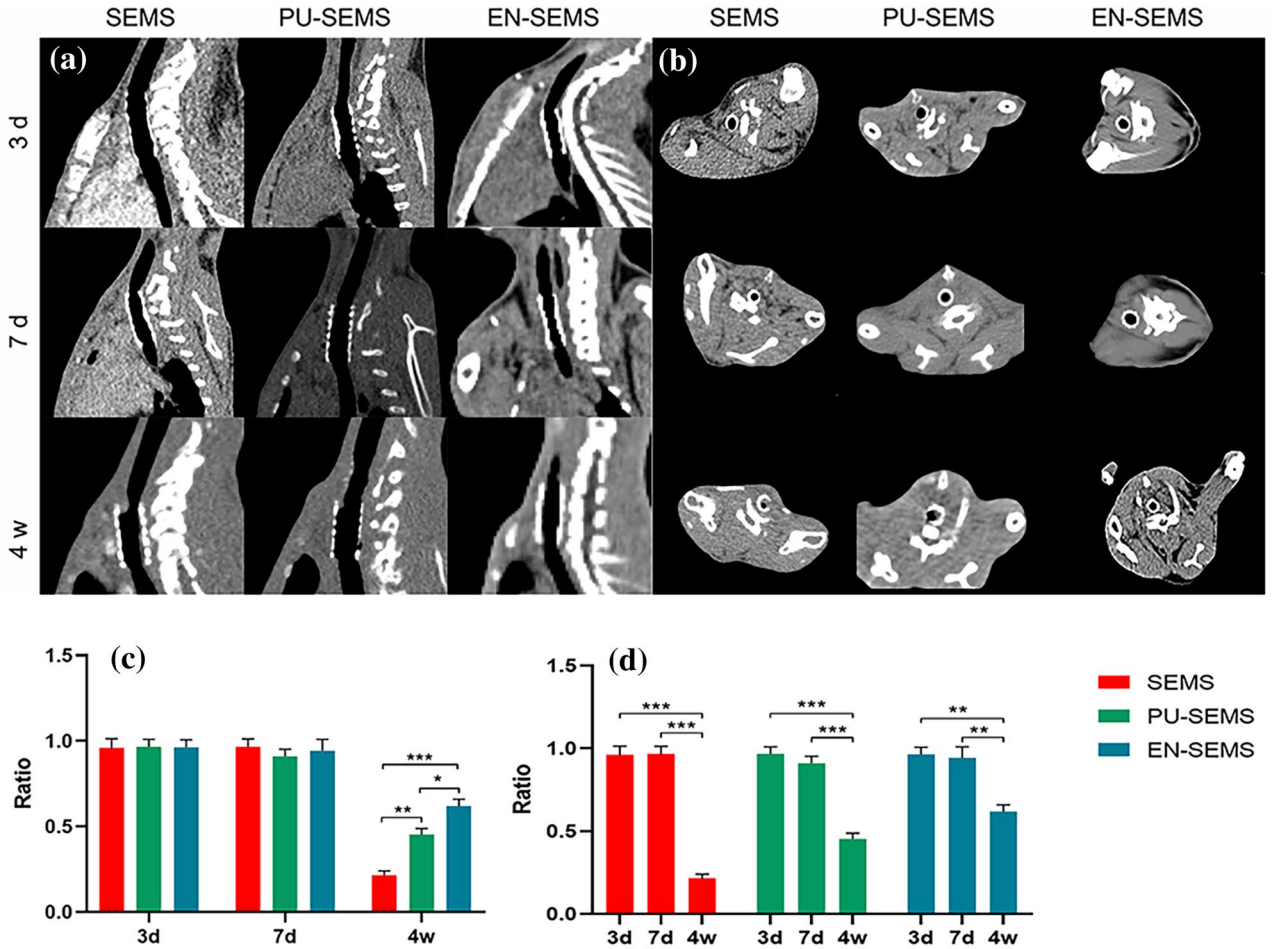
CT image and analysis. (**a**) Sagittal reconstruction computed tomography images after stent placement. (**b**) Axial CT images after stent placement. (**c**,**d**) analysis of tracheal ventilation area between-group and within-group. P-values < 0.05 (*), < 0.01 (**), < 0.001 (***), and < 0.0001 (****).

Representative computed tomography (CT) images of rabbits 3 days, 7 days and 4 weeks after stent placement are shown in Fig. 3a,b. At 3 days post-stenting, the CT examination revealed the fully expandable NF-SEMS, which was similar to the SEMS and PU-SEMS. At 7 days post-stenting, the walls of the three kinds of stents clung to the tracheal wall, with no obvious difference on CT imaging findings. Four weeks after stent placement, granulation hyperplasia was observed in all three groups. In the SEMS group, restenosis occurred in both the proximal and distal parts of the trachea. The granulation tissue grew through the stent mesh and made the stent embedded. In contrast, in the PU-SEMS and EN-SEMS groups, the covered membrane prevented the growth of granulation tissue. Furthermore, stent distal migration occurred in one case in the PU-SEMS group 4 weeks after stenting. No stent migration occurred in the SEMS and EN-SEMS groups.

After stenting 3 days, the TVA ratio of SEMS, PU-SEMS and EN-SEMS was 0.959 ± 0.044, 0.966 ± 0.035 and 0.964 ± 0.035, respectively. There were no between-group differences (p > 0.05). After stenting 7 days, the TVA ratio of SEMS, PU-SEMS and EN-SEMS was 0.966 ± 0.037, 0.910 ± 0.035 and 0.943 ± 0.054, respectively. There were no between-group differences (p > 0.05). After stenting 4 weeks, the TVA ratio of SEMS, PU-SEMS and EN-SEMS was 0.214 ± 0.021, 0.453 ± 0.028 and 0.619 ± 0.033, respectively. There were significant between-group differences (p < 0.05). All the 3 groups with significant within-group differences. The comparative analysis suggested that the difference between post-stenting 3 days and 4 weeks with statistical significance. Also, between 7 days and 4 weeks (Fig. 3c,d).

### Histological findings

**Figure 4:**
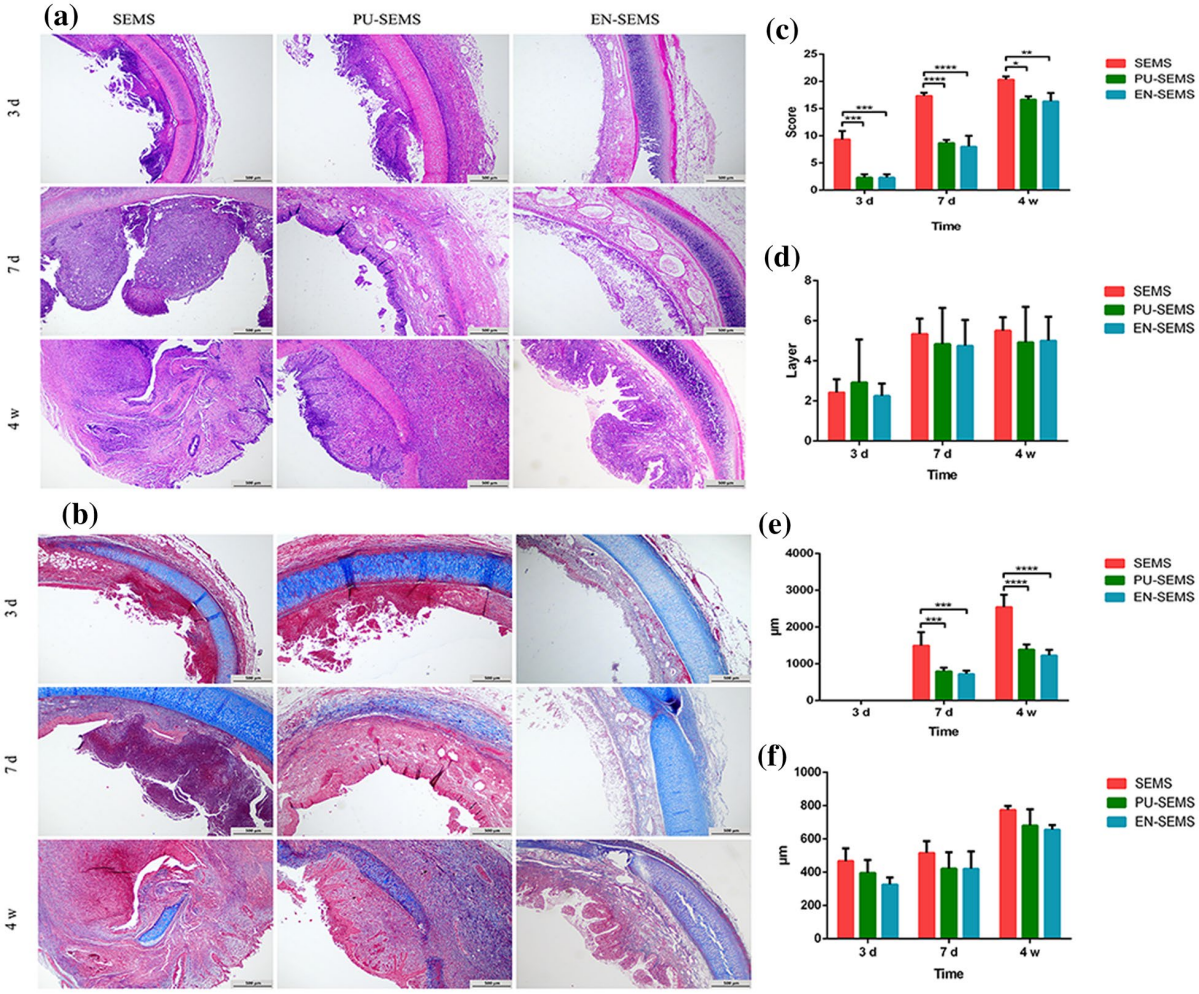
Pathological images and analysis. (**a**) Representative images of tracheal samples submitted to haematoxylin–eosin staining and (**b**) Masson’s trichrome staining. (**c**) Stented tissue scores in the SEMS group were higher than both the PU-SEMS and EN-SEMS groups 3 days, 7 days and 4 weeks at post-stenting. (**d**) There was no statistically significant difference in the number of epithelial layers at 3 days, 7 days and 4 weeks post-stenting. (**e**) No papillary projection formation 3 days after stent placement. The thickness of papillary projection in the SEMS group was significantly higher than in the PU-SEMS and EN-SEMS groups at 7 days and 4 weeks post-stenting. (**f**) The thickness of submucosal fibrosis in the SEMS, PU-SEMS and EN-SEMS groups at 3 days, 7 days and 4 weeks post-stenting, with no statistical difference. P-values < 0.05 (*), < 0.01 (**), < 0.001 (***), and < 0.0001 (****).

Representative HE and MTS staining images are shown in Fig. 4a,b. The mean stented tissue scores in the SEMS group were higher than those in both the PU-SEMS and EN-SEMS groups at post-stenting 3 days (9.33 ± 1.53 vs 2.33 ± 0.58, p < 0.001; 9.33 ± 1.53 vs 2.33 ± 0.58, p < 0.001), 7 days (17.33 ± 0.58 vs 8.67 ± 0.58, p < 0.0001; 17.33 ± 0.58 vs 8.00 ± 2.00, p < 0.0001) and 4 weeks (20.33 ± 0.58 vs 16.67 ± 0.58, p < 0.05; 20.33 ± 0.58 vs 16.33 ± 1.53, p < 0.05) (Fig. 4c). However, among the three groups of SEMS, PU-SEMS and EN-SEMS, there were no statistically significant differences in the mean number of epithelial layers at post-stenting 3 days (2.42 ± 0.67 vs 2.92 ± 2.15 vs 2.25 ± 0.62), 7 days (5.33 ± 0.78 vs 4.83 ± 1.80 vs 4.75 ± 1.29) and 4 weeks (5.50 ± 0.67 vs 4.92 ± 1.78 vs 5.00 ± 1.21) (Fig. 4d). At post-stenting 3 days, the pathological findings suggested that there was no papillary projection formation. The thickness of papillary projection in the SEMS group was significantly higher than in the PU-SEMS and EN-SEMS groups at post-stenting 7 days (1496.33 ± 361.56 μm vs 794.33 ± 100.80 μm, P < 0.001; 1496.33 ± 361.56 μm vs 720.33 ± 91.13 μm, P < 0.001) and 4 weeks (2540.67 ± 341.97 μm vs 1388.33 ± 141.62 μm, P < 0.0001; 2540.67 ± 341.97 μm vs 1229 ± 153.80 μm, P < 0.0001) (Fig. 4e). The thicknesses of submucosal fibrosis in the SEMS, PU-SEMS and EN-SEMS groups at post-stenting 3 days were 466.33 ± 77.69 μm vs 395.33 ± 78.65 μm vs 325.67 ± 42.67 μm, at post-stenting, at post-stenting 7 days were 515.33 ± 70.59 μm vs 421.33 ± 98.17 μm vs 420.00 ± 104.40 μm, and at post-stenting 4 weeks were 774.00 ± 24.52 μm vs 680.67 ± 97.04 μm vs 655.00 ± 27.84 μm. No statistically significant difference was noted among the three groups at any of the three time points (Fig. 4f).

## Discussion

In this study, an EN-SEMS was fabricated and compared with SEMS and PU-SEMS in rabbit trachea. The main findings were as follows: (i) the expansion of EN-SEMS was similar with PU-SEMS and SEMS. (ii) the granulation tissue formation in SEMS group was more severe than in the PU-SEMS and EN-SEMS groups at 7 days and 4 weeks post-stenting; and PU-SEMS group was more severe than EN-SEMS at 4 weeks post-stenting. (iii) the stented tissue score in the SEMS group was higher than those in the PU-SEMS and EN-SEMS groups at 3 days, 7 days and 4 weeks post-stenting. However, the difference between the PU-SEMS and EN-SEMS groups was not significantly different at 3 days, 7 days and 4 weeks post-stenting. Compared with uncovered SEMS, the application of covered stent, including PU-SEMS and EN-SEMS, was associated with less severe stent-induced injury. EN-SEMS are better than PU-SEMS at 4 weeks post-stenting.

Drug-eluting stents (DESs) are promising, combining the functions of support and local treatment. Currently, bare SEMS with polymer-covered wire comprises the main structure of DESs^3^. Others include biodegradable polymer stents and reservoir gelatine gel system stents^8,11^. However, the degradation of polymer induced a local inflammatory reaction, which may contribute to granulation hyperplasia^12^. In our study, poly-L-lactide-caprolactone (PLCL), a biodegradable polymer with good biocompatibility, was chosen as the material for electrospun nanofibre membrane fabrication. The nanofibre membrane made by electrospinning has a multi-void structure and a large specific surface area, making it a good potential carrier for drug release. However, the effect of the PLCL nanofibre membrane on granulation hyperplasia still needs to be explored. Thus, we explored and scored the stent-related trachea injury after the placement of SEMS, PU-SEMS and EN-SEMS in rabbit trachea.

Stent placements in a healthy luminal organ to explore stent-induced tissue hyperplasia have been successfully performed in trachea^13,14^, oesophagus^15^ and urethra^16,17^. The candidate animal models include rabbit, pig, canine and rat. However, the diameter of rabbit trachea is similar to that of human children. In our study, the placement of SEMS, PU-SEMS and EN-SEMS under the guidance of fluoroscopy in rabbit trachea was feasible. Stent migration did not occur in any of the 27 rabbits. The stents size in this study was 20 mm in length and 8 mm in diameter. Stents of this size have enough radical force to cause mechanical injury to the trachea. The wound healing response to the injury includes inflammation, proliferation and remodelling^16^. The whole process of wound healing lasts almost four weeks. The inflammation phase begins immediately after stent placement, and the proliferation phase begins at 4 days post-stenting. Thus, we chose 3 day, 7 days and 4 weeks post-stenting as the time points to evaluate the pathological findings.

The pathological findings of the trachea simply indicated that EN-SEMS is similar to PU-SEMS and better than bare SEMS. The pathological images at 3 days, 7 days and 4 weeks post-stenting represent the inflammation, proliferation and remodelling phases of wound healing. The stented tissue score and thickness of the papillary projection in the SEMS group were higher than those in the PU-SEMS and EN-SEMS groups. The difference was statistically significant. These results can be explained by the structure of a covered stent vs a bare stent. A covered stent has the potential to prevent granulation tissue growth through the stent mesh and reduce the stressed epithelium area caused by wire. The differences between the PU-SEMS and EN-SEMS groups were not statistically significant. These results indicate that the membranes of EN-SEMS are similar to those of PU-SEMS.

There are two limitations in this study. First, all three kinds of stent were placed in healthy rabbit trachea, and the wound healing process after stent placement may differ from that of benign tracheal stenosis. Second, the duration time of EN-SEMS in the trachea was not sufficient to observe the degradation of the PLCL electrospun nanofibre.

## Conclusion

In conclusion, the stent-induced granulation tissue formation in EN-SEMS is less severe than that of PU-SEMS and SEMS. EN-SEMS has smaller radial force, and the tracheal ventilation ratio after stent placement better than that of PU-SEMS. EN-SEMS is a good candidate for drug-eluting stent fabrication.

## Data Availability

The data used to support the findings of this study are available from the corresponding author upon request.
